# Compressive Sensing Imaging Spectrometer for UV-Vis Stellar Spectroscopy: Instrumental Concept and Performance Analysis

**DOI:** 10.3390/s23042269

**Published:** 2023-02-17

**Authors:** Vanni Nardino, Donatella Guzzi, Cinzia Lastri, Lorenzo Palombi, Giulio Coluccia, Enrico Magli, Demetrio Labate, Valentina Raimondi

**Affiliations:** 1 IFAC-CNR, 50019 Sesto Fiorentino, Italy; 2 Politecnico di Torino–DET, 10129 Torino, Italy; 3 Leonardo-Company S.p.A., 50013 Campi Bisenzio, Italy

**Keywords:** compressive sensing, stellar spectroscopy, spatial light modulator, DMD, imaging spectrometer

## Abstract

Compressive sensing (CS) has been proposed as a disruptive approach to developing a novel class of optical instrumentation used in diverse application domains. Thanks to sparsity as an inherent feature of many natural signals, CS allows for the acquisition of the signal in a very compact way, merging acquisition and compression in a single step and, furthermore, offering the capability of using a limited number of detector elements to obtain a reconstructed image with a larger number of pixels. Although the CS paradigm has already been applied in several application domains, from medical diagnostics to microscopy, studies related to space applications are very limited. In this paper, we present and discuss the instrumental concept, optical design, and performances of a CS imaging spectrometer for ultraviolet-visible (UV–Vis) stellar spectroscopy. The instrument—which is pixel-limited in the entire 300 nm–650 nm spectral range—features spectral sampling that ranges from 2.2 nm@300 nm to 22 nm@650 nm, with a total of 50 samples for each spectrum. For data reconstruction quality, the results showed good performance, measured by several quality metrics chosen from those recommended by CCSDS. The designed instrument can achieve compression ratios of 20 or higher without a significant loss of information. A pros and cons analysis of the CS approach is finally carried out, highlighting main differences with respect to a traditional system.

## 1. Introduction

Space application domains are increasingly experiencing the generation of very large amounts of data, which can sometimes hinder their full exploitation. If, on one hand, a high information content of the data is required for either scientific or commercial exploitation, on the other, there is the need to limit the payload budget as much as possible also by reducing the amount of data to be handled on board. The handling of a large amount of data, in fact, requires not only significant onboard resources to be used for data storage and processing but also involves considerable downlink requirements. In actuality, the payload should be able to provide high data rates—yet with limited power consumption—and an adequate transmission system that otherwise can become a bottleneck.

Compressive sensing (CS) has been proposed as a possible approach that can contribute to mitigating these issues via the acquisition of natively compressed data and through its capability to perform onboard data processing for information extraction at little computational cost by screening and selecting the acquired images prior to their reconstruction. For this reason, in recent years, several research studies were also funded by the European Space Agency (ESA) in order to investigate the potential of CS in diverse space application domains.

CS is a novel data acquisition technique that leverages the feature of many natural signals of being highly correlated. A high correlation entails the existence of a domain (integral transform) in which the signal is sparse and only a small fraction of the transform coefficients is significantly different from zero [1,2,3]. CS theory affirms that an exactly sparse signal can be sensed with a small number of linear measurements without any information loss [4]. As a consequence, the number of samples foreseen using the Nyquist–Shannon sampling theorem for the reconstruction of a generic signal could, in the appropriate transformed domain, be reduced without any loss of information. If the signal is only approximately sparse, then the reconstruction is approximate. The signal could then, in principle, be reconstructed by acquiring only few measurements, hence the term CS, meaning that the signal is compressed at the acquisition stage.

From the instrumental point of view, an interesting feature of the CS approach is the possibility of using a single-pixel detector instead of a two-dimensional (2D) array detector to acquire an image. Based on a time-multiplexing technique, a single-pixel camera is able to acquire an image using a single photodetector element instead of an array of detectors. This feature becomes particularly interesting when dealing with spectral ranges such as ultraviolet (UV) and infrared (IR), for which matrix detectors are very expensive or available only with a small number of elements. A simple scheme for such technique is offered by the iterative acquisition of a multiplexed (i.e., spatially integrated) binary-coded image that is obtained by superimposing an on-off mask; spatial coding produces multiplexed (but independent) measurements of the image. In principle, an N × M pixels image can be reconstructed (without any compression) using N × M independent measurements and the corresponding binary masks; differently, if we use a smaller number of measurements, it will be reconstructed with intrinsic compression. The latter approach is the one followed by the CS techniques. The implicit hypothesis is that the image is sparse in some basis. Thus, the information loss caused by the missing samples can be neglected. This implies that image compression is performed at sampling stage. 

The CS paradigm has enabled the development of novel instruments, such as the single-pixel camera developed by the Rice University [5]. Following the development of the single-pixel camera, other similar prototypes were implemented in different laboratories [5,6,7]. CS theory has also been applied for the development of three-dimensional (3D) cameras [8,9] and of hyperspectral imaging systems [3,10,11,12]. The results of these studies have shown some advantages but also several issues, such as the unavailability of high-speed modulators [9,10,11]. The interest in developing hyperspectral systems with CS technology was primarily fed by the opportunity of reducing the data throughput. The Georgia Institute of Technology developed a CS architecture that employs a standard 256 × 256 CMOS: the sensor and its interface were combined with a complex computational circuitry that performs the domain transformation intrinsic in a CS-system instead of employing a light modulator in front of a single-pixel detector [13]. Another study has been conducted in the frame of the Compressive Optical MONTAGE Photography Initiative (COMP-I), funded under DARPA’s MONTAGE program, with the aim of producing a miniaturized visible (VIS) and longwave infrared (LWIR) camera [14,15]. CS approach was also used for VIS and infrared (IR) microscopy [16,17]. Several studies have been performed to better understand the potentiality of CS in the medium-wave infrared (MWIR) spectral range [18,19,20]. 

Concerning the space application domains, few studies have addressed the investigation of the potential of CS for Earth observation (EO) applications [21,22,23,24,25,26]. Among these, an ESA-funded study for the development of a laboratory demonstrator of a CS-based hyperspectral imager for Earth Observation [23] which highlighted some critical issues, such as stray light control and the need of very high-speed light modulator, can be mentioned.

The major advantages that can be expected from implementing CS-based instrumentation for space applications can be summarized as follows:

- Use of detector arrays with a reduced number of pixels (down to a single pixel, as in the case of the single-pixel camera), which makes the implementation of imagers in spectral regions with reduced availability of large focal-plane arrays easier;
- Inherent compression of data, which is a typical feature of the CS paradigm and can impact the reduction of data throughput;
- Onboard processing at little computational cost.

In space instrumentation, these features assume additional specific meaning. Inherently compressed data implies that the compression board is not needed any longer, which can contribute to reducing the mass, volume, and power budgets to some extent and also to relaxing downlink requirements. In addition, the use of a single-pixel detector can be particularly captivating in those spectral ranges in which large focal plane arrays are very expensive or do not exist at all. CS-enabled inherent compression offers further advantage for the observation of intrinsically sparse scenes, and this particularly applies to slitless spectroscopy, which deals with inherently spectrally multiplexed images. The idea is that the images of punctual targets (i.e., of a single star or a multistellar field) can be spectrally split by using a dispersive medium in their spectral components along a specific direction. If the targets are well apart in the image, the split spectra will not superimpose on each other, allowing the detection of both the spectral characterization of the radiance of each target and its position in the image. 

In this paper, we present the feasibility study of an elegant breadboard of a CS-based imaging spectrometer for slitless stellar photometry. The proposed instrumental concept uses a digital micromirror device (DMD) as a binary-coding mask—applied to the image focused by the collection optics—in order to implement a CS single-pixel architecture. This is the first study in the literature to investigate the feasibility of such an instrument and to assess the expected performances, while discussing main advantages and also drawbacks. The study was performed in the frame of the “Optical Compressive Sensing Technologies for Space Applications” (OCS-TECH) project funded by the ESA. The main goal of the project was to investigate the potential of CS-based optical instrumentation for space applications in several application domains, namely EO, planetary exploration, and space science (SS).

## 2. Theoretical Background

CS is based on the concept of sparsity. The information carried by a continuous “sparse” signal may be much smaller than suggested by its bandwidth or, in the discrete-space domain, the samples acquired by a 2D discrete sensor array are redundant. The benefit of CS is that it permits the design of efficient sensing protocols that capture the signal in a very compact fashion while retaining and allowing the recovery of the useful information contained in a sparse signal. 

In practice, natural signals may not be exactly sparse, but they are usually said to be “compressible”. A multidimensional signal with a strong average spatial and/or spectral autocorrelation is represented in a redundant fashion via a conventional sensing system. For compressible signals, however, a mathematical representation—usually a linear transform—of the signal exists, in which the only few coefficients are significantly different from zero. CS exploits this by sensing the signal through a small set of random projections, from which the signal can be reconstructed, at least approximately. CS is said to be a “universal” sensing paradigm in that the transform is not employed during acquisition but only during image reconstruction, enabling signal reconstruction from the acquired set of measurements. The linear measurements are usually taken by modulating the image with random coefficients; hence, they are also denoted as “random projections”. When radiometric and spectroscopic signals are considered, an optical device such as a spatial light modulator (SLM) would be the natural choice for optically computing such random projections. 

More in-detail, CS represents a signal through a small set of linear measurements:

*y* = *Φ*
*x*+ *η*, (1)

where *x* is the signal of interest (or an image, e.g., read in raster order), *Φ* is a sensing matrix (whose entries are typically drawn at random), *y* is a vector of linear measurements having many fewer entries than *x*, and *η* is a noise vector. If the signal *x* is compressible, then nonlinear reconstruction algorithms can recover *x* exactly or with high fidelity. Compressibility usually requires representing the signal in a different domain using, e.g., a discrete cosine transform or discrete wavelet transform as:

*x* = *Ψc*, (2)

with $\Psi^{-1}$ being the linear operator sparsifying the signal and *c* a vector of transform coefficients. CS is typically performed in the natural image domain, whereas reconstruction aims to recovering an approximation c* ≃ c from which one can recover an approximation of the signal through the corresponding inverse transform as:

(3) $$x^{*}=\Psi c^{*}\;\mathrm{and}\;x^{*}≃x$$

The problem of recovering *x* from knowledge of *y* is that Φ (and Ψ) is underdetermined and has infinitely many solutions. Nonlinear reconstruction algorithms can be written, e.g., choosing the sparsest solution from among all possible solutions, i.e.:

(4) $$x^{*}=\min∥x{∥}_{0}\;s.t.\;∥y-\Phi x^{*}{∥}_{2}<\varepsilon$$

The reconstruction problem is often recast as a convex problem, replacing the l0 pseudonorm with the l1 norm. An alternate solution consists of employing greedy iterative algorithms, which are often based on alternate gradient steps, to enforce the solution to satisfy:

(5) $$∥y-\Phi x^{*}{∥}_{2}<\varepsilon ,$$

And thresholding steps to obtain an approximately sparse solution.

## 3. Working Principle of a CS-Based Instrument

The working principle of a CS instrument relies on the acquisition of a series of “measurements” obtained as the scalar product of the signal with a pseudorandom sequence. In practice, this is obtained by using a SLM that physically performs the scalar product between a random pattern and the incoming light followed by an optical assembly that concentrates the signal onto a single-element detector. The reconstruction of the signal requires the determination of the sparsest signal that matches the available measurements, which can be performed by using, for example, linear programming techniques.

**Figure 1:**
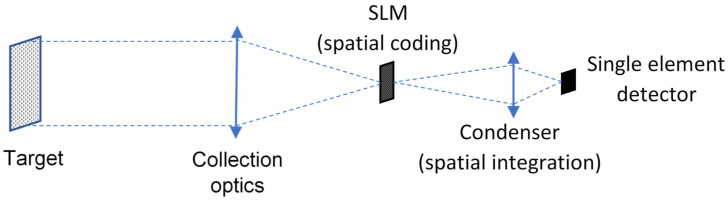
Working principle of a CS-based instrument.

Figure 1 shows a simplified block diagram illustrating the working principle of a CS instrument: the scene of interest (target) is collected by a suitable optics and focused on the image plane field stop at the SLM plane. This device performs the scalar product by applying a spatial binary coding mask to the image. This coding is obtained by setting the SLM elements in a specific configuration of on/off states. If the device used as SLM is a DMD, this means that each of the DMD micromirrors is set either in an “on” or an “off” state, providing an on–off masking of the scene. The whole coded image is collected by a condenser and fed to the detector for signal acquisition: the latter step corresponds to performing a spatial integration of the coded image and subsequent acquisition. The iteration of this procedure allows the collection of a series of spatially integrated measurements of the same scene, each measurement corresponding to the scene coded by a different mask. The use of a suitable basis of coding masks allows the reconstruction of the image. The acquisition and use of a limited number *K* of measurements—with *K* being far less than the number *N* of pixels of the reconstructed image—allows the reconstruction of the original image in a sparse domain, i.e., the reconstruction of an intrinsically at-sampling-time-compressed image by using ad hoc algorithms.

## 4. The CS Based UV-Vis Imaging Spectrometer for Stellar Spectroscopy

The CS based instrument proposed in this paper is a slitless imaging spectrometer for SS applications operating in the UV-Vis spectral range. The instrumental concept was inspired by the Space Telescope Imaging Spectrograph (STIS) and Imaging and Slitless Spectroscopy Instrument for Surveys (ISSIS) instruments, which were developed for photometric astronomy.

In the following sections, we will first provide an overview of the UV-Vis spectrometers for SS applications operating aboard ongoing missions or under study for future missions with an analysis of the sparsity grade of the relevant data in order to preliminarily assess the suitability of the CS paradigm. Secondly, we provide an overview of the proposed CS-based instrument’s optical architecture. Thirdly, we describe the optical design of the instrument with the analysis of the relevant optical performances. Finally, we describe the procedures adopted to simulate the data acquired by the instrument and we evaluate the performance obtained in the final reconstruction of the images by using ad hoc reconstruction algorithms.

### 4.1. Instrumental Heritage and Application Domain

Presently, there are only two instruments working in the UV-Vis: the STIS instrument onboard the Hubble Space Telescope [27] and the Blue Photometer on board the GAIA satellite [28]. Other instrumental concepts were proposed and subsequently abandoned (e.g., ISSIS [29]).

There are several examples of spaceborne hyperspectral imagers operated onboard past and ongoing missions and working in the UV-Vis spectral range. These are:

- UVIS (Ultraviolet Imaging Spectrograph) on Cassini: this has been designed to measure UV light in the spectral range from 55.8 nm to 190 nm. As an example, UVIS has permitted the determination of the composition, distribution, aerosol particle content, and temperatures of the atmospheres of Saturn and Titan [30];
- STIS instrument, aboard the Hubble Space Telescope: STIS is an imaging spectrograph providing spatially resolved spectroscopy in UV-Vis, high spatial resolution echelle spectroscopy in UV, solar-blind imaging in UV, and direct and coronagraphic imaging in Vis;
- Galaxy Evolution Explorer (GALEX): an orbiting space telescope for the observation of galaxies in UV light. GALEX was launched on 28 April 2003 and operated until early 2012 [31];
- Blue Photometer on GAIA, whose spectral dispersion is a function of wavelength and varies from ~3 to ~27 nm/pixel covering the wavelength range ~330–680 nm [32];
- ISSIS instrument for WSO (World Space Observatory). The baseline for the ISSIS design consists of two acquisition channels: a far ultraviolet (FUV) channel covering the 115 nm–175 nm spectral range and a near ultraviolet channel (NUV) in the 185 nm–320 nm spectral range.

The proposed CS instrument is mainly inspired to the STIS and ISSIS instruments, which were developed for photometric astronomy. Its operation is limited either to long-slit spectroscopy, such as in the STIS instrument, or to stellar photometry. The first application is more critical due to requirements in terms of data quality and pointing and platform stability: however, a scanning mirror feeding the dispersive element could be envisaged in order to enhance the observation capabilities of the instrument.

In order to provide a first evaluation of the expected data quality and compression ratio (CR), we selected and analyzed a set of data from the online archive with spectroscopic data from the STIS on the Hubble Space Telescope [33]. The image sparsity was evaluated in various domains: the original domain of the pixel values and the 2D and 3D discrete cosine transform domains. The sparsity level—defined as the percentage of coefficients containing at least 99% of the signal energy—is reported in Table 1. Since the image was quite noisy, we also considered a thresholded image to assess the sparsity, which showed that the image was indeed sparse in the original domain after thresholding.

### 4.2. Instrumental Architecture 

**Figure 2:**
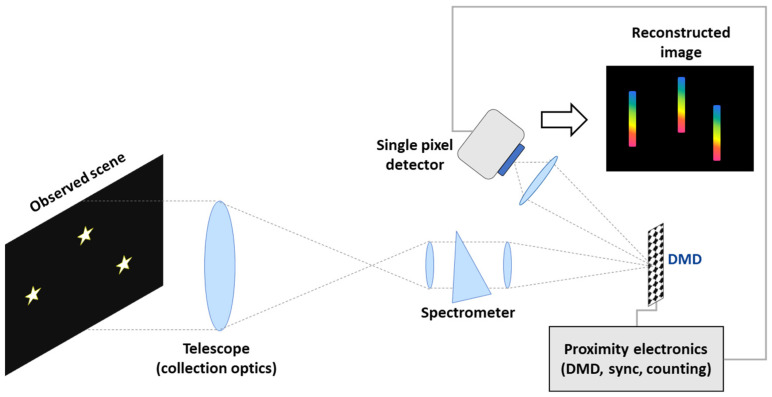
CS-based imaging spectrometer: block diagram.

Figure 2 shows an overall simplified block diagram of the instrument: the core section is the optical sub-system, which includes optical components like lenses and prism, the DMD used as SLM and the PhotoMultiplier Tube (PMT) module for photon counting. The proximity electronics instead contain the DMD configuration board, the counting unit, the signal synchronization unit, and an electronic board for temperature control and regulation. A data handling/processing unit (PDHU) and power supply must be also foreseen (not detailed in the diagram).

The main driving parameters for the definition of the system specifications were: the DMD technical specifications, single-pixel detector specifications (with particular reference to its ability in detecting low level signals), and the requirements in terms of integration time (for which we used the same range of the STIS instrument). We selected all commercial off-the-shelf (COTS) components. The DMD was chosen from among the DLP^®^ models manufactured by the Texas Instruments. The DMD key parameters were micromirror pitch, micromirror tilt angle, modulation speed, and compatibility with future space qualification. 

The optical section of the CS payload is made up of the following main elements (Figure 2): a telescope, an imaging spectrometer, a DMD used as SLM, a condenser lens, a single element detector. The optical design was performed by considering a modular structure for the different optical components.

The telescope is the entrance optics of the payload. The telescope is a Schmidt–Cassegrain telescope designed with a parabolic surface for the primary mirror and hyperbolic surface for the secondary mirror. The selected material was coated aluminium since it has an improved transmittance in the UV–Vis spectral range and is compatible with the space environment. A folding mirror feeds the image produced by the telescope to the input of the imaging spectrometer. The spectrometer assembly is a prism-based spectrometer designed for performing slitless measurements. The assembly is composed of collimating optics, a prism, and imaging optics. The instrument was designed to be pixel-limited in the entire 300 nm–650 nm spectral range with spectral sampling that varies depending on the central wavelength (2.20 nm@300 nm; 18.1 nm@600 nm; 22.0 nm@650 nm).

The functioning is as follows: the incoming signal is fed to the spectrometer, which is used to produce a spectral image of the observed section of the sky. The DMD implements the CS coding by spatially modulating the incoming light when a binary coding mask is applied to the DMD micromirrors. The DMD reflects the coded image to a condenser lens. The condenser lens concentrates the image on a single-element detector, thus performing a spatial integration of the CS-coded image. The single-pixel sensor is a PMT module. The latter works in photon counting mode due to the small number of photons (photoelectrons) that reach the sensor during each CS measurement. The expected flux of radiation reaching the DMD and PMT during each measurement was numerically simulated by considering a realistic scenario, as explained in detail in Section 5.3. 

The proximity electronics supervises DMD coding operation, counts the pulses coming from PMT module, and synchronizes the generation of the CS masks on DMD with the counting operation. 

### 4.3. Optical Design

**Figure 3:**
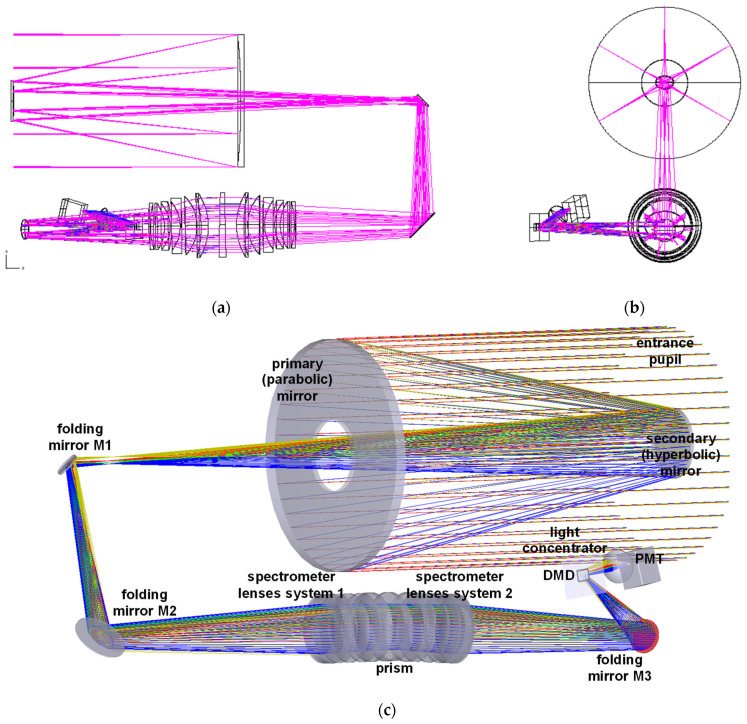
Optical design of the optical section: (**a**) side view, (**b**) front view, and (**c**) 3D rendering of the entire optical system.

For the optical design (Figure 3) of the system, we used a modular structure. The system is made of the following parts: a collection optics, a spectrometer, a DMD for masking the image plane, and a condenser that performs the spatial integration of the modulated image. The spectrometer can be further divided into: collimator, prism, and focuser (symmetrical to the collimator). The spatial integrator is implemented by using a condenser lens acting as light concentrator that focuses the integrated signal onto the single-pixel detector.

A Cassegrain-type reflector telescope ((Figure 3a) is used as the collection optics. We chose this type for its capability of minimizing the field curvature. An almost-flat image is needed for applying the masking to the image on the DMD plane. Refractor elements are not used in the telescope, being a mere reflector telescope made of a primary parabolic mirror and a secondary hyperbolic mirror. The focus is beyond the primary surface. The two mirrors are mounted in line with respect to the optical axis: the primary mirror has a circular aperture corresponding to the zone obscured by the secondary mirror (Figure 3b).

The main purpose of the folding mirrors is to make the instrument more compact.

It is worth to note that the optical system has a nonplanar design due to the fact that the DMD micromirrors rotate along a diagonal axis. This adds to the reflected direction a 45° rotation out of the plane formed by the telescope and spectrometer optical axes (Figure 3a). 

Table 2 reports all the main specifications of the instrument with the technical data of the main optical components.

**Figure 4:**
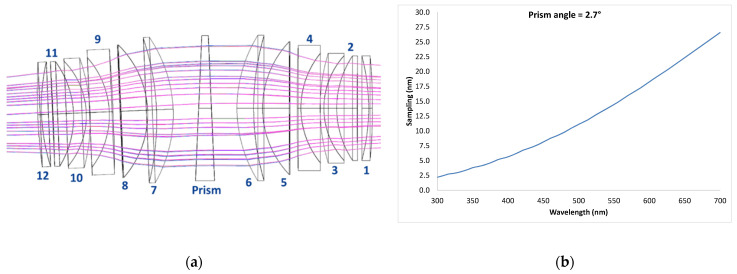
Optical design of the CS instrument: (**a**) spectrometer layout and (**b**) corresponding spectral sampling curve.

The optical system and the prism were designed in order to reach the best optical performance in terms of modulation transfer function (MTF) and point spread function (PSF) on the DMD image plane. At the same time, the optical materials were also selected in order to improve the transmittance in the UV–Vis spectral range, also taking into account their compatibility for their use in space environment. The optical performances on the DMD image plane were improved by finding the focal length of the spectrometer and angle of dispersion of a single prism ad hoc. Additionally, due to the optical characteristics of glasses (low transmittance) in the 250 nm–300 nm range, the operational spectral range of the system was set to 300 nm–650 nm. The spectrometer is a symmetric system of lenses with 723.5 mm effective focal length (BFL494.5 mm). The design relies on a 2.7° triangular-base prism in silica glass in order to provide superspectral resolution, spreading the 300–650 um band on 50 pixels. The materials were selected in order to minimize the aberrations of the entire system and to improve the optical transmittance in the UV. Figure 4 shows the layout of the spectrometer together with the spectral sampling curve from 300 nm to 700 nm. 

Table 3 reports all the optical components of the spectrometer and the relevant materials.

**Figure 5:**
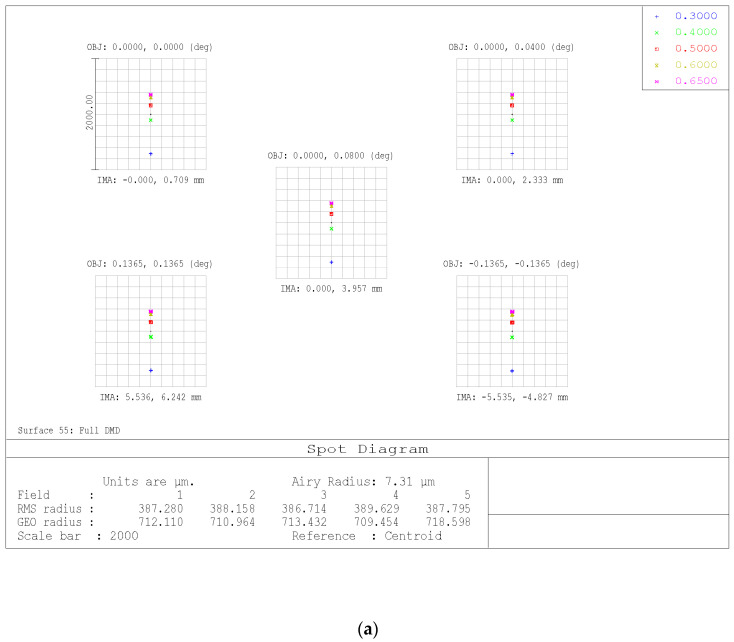

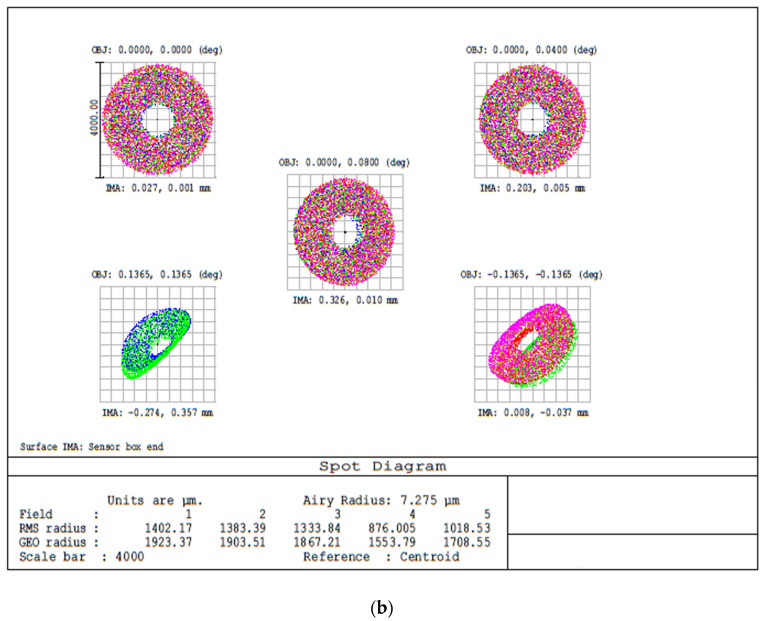
Optical design of the CS instrument: (**a**) spectrum on the image plane (DMD surface) for different angles in the instrument field of view and (**b**) spot radius on the PMT. The PMT is placed where the focused beam diameter is minimal, providing both spot minimization and image mixing on the detector area. The beam at the detector plane is defocused so that the figure shows the corresponding pupil obscuration.

The spatial modulation is performed at the DMD plane by means of applying the coding mask to the micromirrors. Each mask is made of a binary pattern of ON and OFF positions to be applied to the DMD micromirrors. The DMD micromirrors set to the ON position reflect the light towards the condenser. The DMD micromirrors set to the OFF position reflect the light away from the main optical path in order to be dumped. The selected DMD is one of the models produced by Texas Instrument and was designed for operation in UV—specifically, the DLP^®^9500UV model. It features an array dimension of 1920 × 1080 aluminium mirrors, each with a pitch of 10.8 μm. The DMD window transmittance is higher than 85% in the spectral range of interest. The spectrum on the image plane (DMD surface) for different angles in the instrument field of view is shown in Figure 5a.

The light condenser, which is a low f/# lens, concentrates the light on the sensitive area of the single-pixel detector. The optimized lens has a focal length of 38 mm and a diameter of 16 mm.

The selected single-pixel detector is a PMT module with an Ultra Bialkali cathode and a sensing area of 8 mm. The spot radius on the sensor plane is 2.10 mm (Figure 5b). The detector is positioned where the focused beam diameter is minimal. Such position does not match exactly with the focal length, so the beam is slightly unfocused. Such unfocused image has the additional effect of guaranteeing a uniform mixing of the image pixels on the sensitive area of the detector, thus avoiding systematic errors.

## 5. Performance Analysis

The performance analysis of the CS instrument was carried out in four steps:

- Analysis of the optical performance of the instrument by assessing the optical computer-aided design (CAD) outputs;
- First-order evaluation of the stray light;
- Simulation of a set of data as acquired by the instrument, starting from a set of images acquired from STIS payload and taking in account the optical main specifications of the CS instrument;
- Reconstruction of the images by applying ad hoc algorithms and by comparing the reconstructed images with the original ones.

### 5.1. Optical Performance

**Figure 6:**
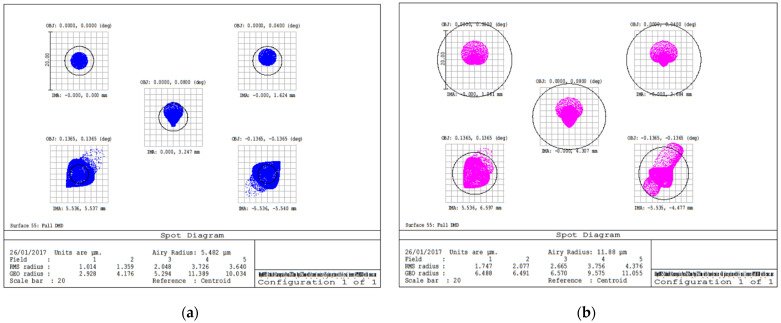
Spot diagram on the image plane (DMD surface) for different angles in the instrument field of view: (**a**) spot diagram at 300 nm, and (**b**) spot diagram at 650 nm.

Figure 6 shows the spot diagram on the image plane (DMD surface) for different angles in the instrument field of view and for different wavelengths. The instrument is almost pixel-limited in the 300 nm–650 nm spectral range. The spectral sampling ranges from 2.2 nm@300 nm to 22 nm@650 nm, with a total of 50 samples for each spectrum. The spectral sampling is similar to that of the GAIA mission.

The system features several baffles to mitigate the effects of stray light along the entire instrument path (see next section), ensuring the protection from internal reflection of the condenser and detector and the trapping of the light reflected by micromirrors in the OFF position.

Optical design was performed using COTS materials—no-space graded glasses were used—although the chosen materials are all compatible with space qualification.

### 5.2. Stray Light Analysis

In CS instruments, stray light mitigation is particularly important since the instrument acquires a fraction (typically, 50%) of the signal, and in addition, a multiplexing approach is adopted. In the case of the proposed instrument, stray light mitigation is even more crucial since the signal received from stars is expected to be weak and the scene is highly sparse. In order to reduce the effects of stray light, we designed a system made of several baffles. 

**Figure 7:**
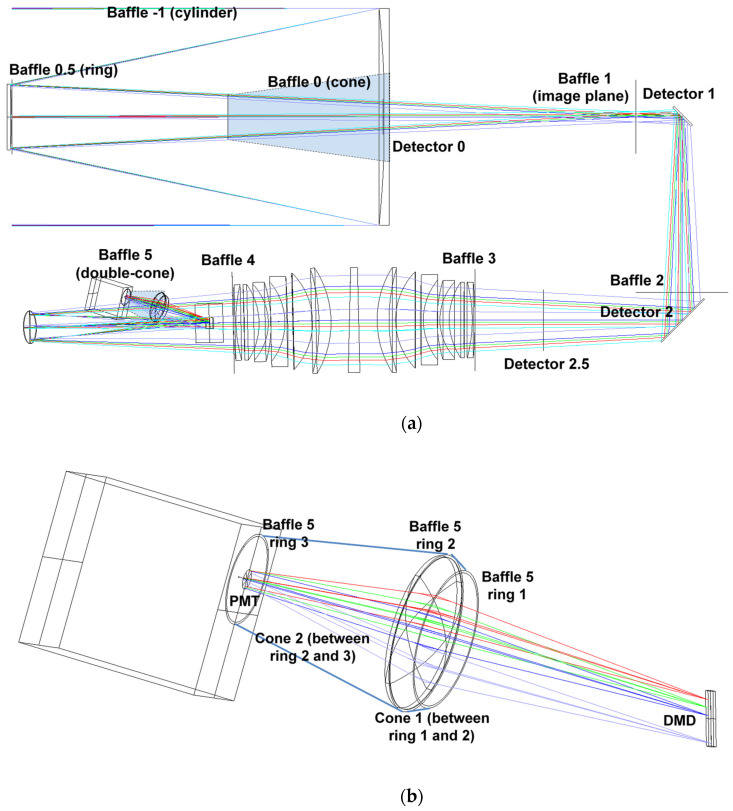
Stray light analysis: (**a**) positions of the baffles and detectors and (**b**) detail of the position and size of Baffle #5.

The zero-order stray light study has led to the positioning and dimensioning of the telescope baffles (Baffles #−1, #0, #0.5 in Figure 7a) on the grounds of geometrical considerations. The positioning and dimensioning of the three telescope baffles were initially tested using the Zemax optical CAD in nonsequential mode by placing a source emitting within an angle of ± 5.5° at the telescope entrance pupil and considering the baffles −1, 0, and 0.5 totally absorbing (zero-order stray light).

In the second step (Figure 7b), we placed another set of baffles between the telescope output and the DMD. We placed four baffles: Baffle #1 on the first image plane, Baffle #2 before the second folding mirror, Baffle #3 at the entrance of the spectrometer group, and Baffle #4 at the end of the spectrometer group. An additional baffle (Baffle #5: double cone shown in Figure 7b) was also placed between the DMD and the PMT.

**Figure 8:**
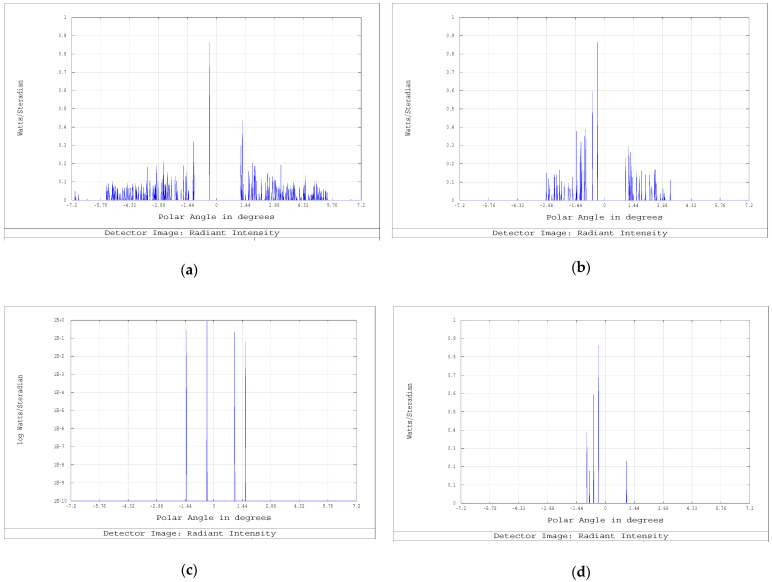
First-order stray light analysis: angular cross sections on the detectors. (**a**) stray light at Detector #0 (after exit pupil, on primary mirror). (**b**) Detector #1 (on the image plane). (**c**) Detector #2 (between the two folding mirrors), (**d**) Detector #2.5 (before the spectrometer).

The subsequent stage consisted of the first-order stray light analysis. The latter took into account the (first-order) scattering of light on the optical surfaces in order to test the baffles efficiency in rejecting residual light flares. The test on the stray light at the first order was made by simulating a source emitting within an angle of ±5.5° at the telescope entrance pupil and by considering Baffles #−1, #0, and #0.5 to be Lambertian reflectors with albedo 5% and Baffles #1 and #2 to be totally absorbing. The photons were simulated by using Zemax optical CAD (in nonsequential mode) and measured by the virtual detectors (Detectors #0, #1, #2, and #2.5 in Figure 7a) to determine the stray light contribution. The results are shown in Figure 8 for all the four detectors: at Detector #0 (Figure 8a), the first-order stray light contribution is still present due to the reflections on Baffle #−1; at Detector #1 (Figure 8b), the stray light is still present, but it does not impinge on the DMD used area since the latter accepts photons only from an angle less than ±0.139°; at Detector #2 and Detector #2.5 (Figure 8c,d, respectively), some residual stray light is still present, but it does not impinge on the DMD used area.

In the whole, the results showed that the designed baffles performed a good stray light suppression along the entire instrument optical path. In particular, this guaranteed the protection from internal reflection of the condenser and of the detector, and the trapping of the light reflected by the micromirrors in the OFF position.

### 5.3. Data Simulation

A number of simulated images were produced and used as input to the CS reconstruction algorithm in order to evaluate the overall system performance. On the whole, three sets of simulated images were generated:

- Reference images: these were ideal images without the system’s MTF, noise, and detector sensitivity;
- Images with a Gaussian MTF: these were used to test the CS reconstruction algorithm with MTF compensation when the MTF had a simple shape and was not wavelength-dependent;
- Images with the system characteristics: these were created by using the optical CAD system features, including spatial–spectral-dependent MTFs.

These sets of images were created by using the same simulation procedure and the following data as inputs:

- Spectrum of stars coming from the STIS spectral library;
- Sky background flux (accounting for earthshine and zodiacal light) as evaluated for STIS;

The procedure we applied for simulating realistic scenarios was as follows. First, we evaluated how many stars (on average) for visual magnitudes (m_v_) from 2 to 11 could fall into the field of view (FOV) of the instrument. To do this, we used the astronomical catalogue of Hipparcos (https://www.cosmos.esa.int/web/hipparcos/catalogues (accessed on 20 December 2022)). The results of this analysis showed that, for the envisaged FOV, we could expect to detect:

0 to 1 stars with m_v_ = 4 to 7.

1 to 3 stars with m_v_ = 7 to 11.

The probability of detecting a star with m_v_ less than 4 is very low (less than 1%) unless we point directly at it. As a consequence, we simulated an image containing three stars’ spectra—one for the class m_v_ from 4 to 7, and two for the class m_v_ from 7 to 11.

We chose three stars from the STIS spectral library belonging to different spectral types.

The spectra acquired from STIS were resampled following the spectral dispersion function of the instrument, which has spectral sampling varying from 1 nm to 20 nm. The data was converted from flux to photon flux. The pixels of the images that did not contain the stars’ spectra had the values of the sky background (accounting for earthshine and zodiacal light) as evaluated for STIS [34], plus normally distributed Gaussian noise to take into account its intrinsic variability.

The obtained image was filtered with the optical MTF of the system, which is wavelength dependent. The lenses and the mirrors of the system yield a signal attenuation of 0.5.

The flux of photons was converted into photons by using the entrance pupil of the telescope, the spectral dispersion function, and an integration time of 11.5 ms (total measurement time 300 s for the acquisition of 512 × 51 measurements, with an equivalent CR of 10).

The average number of photons that reaches the detector is reduced by a factor 0.5 after the DMD due to the binomial modulation. The average number of photons at the detector should be the order of 10^4^. 

**Figure 9:**
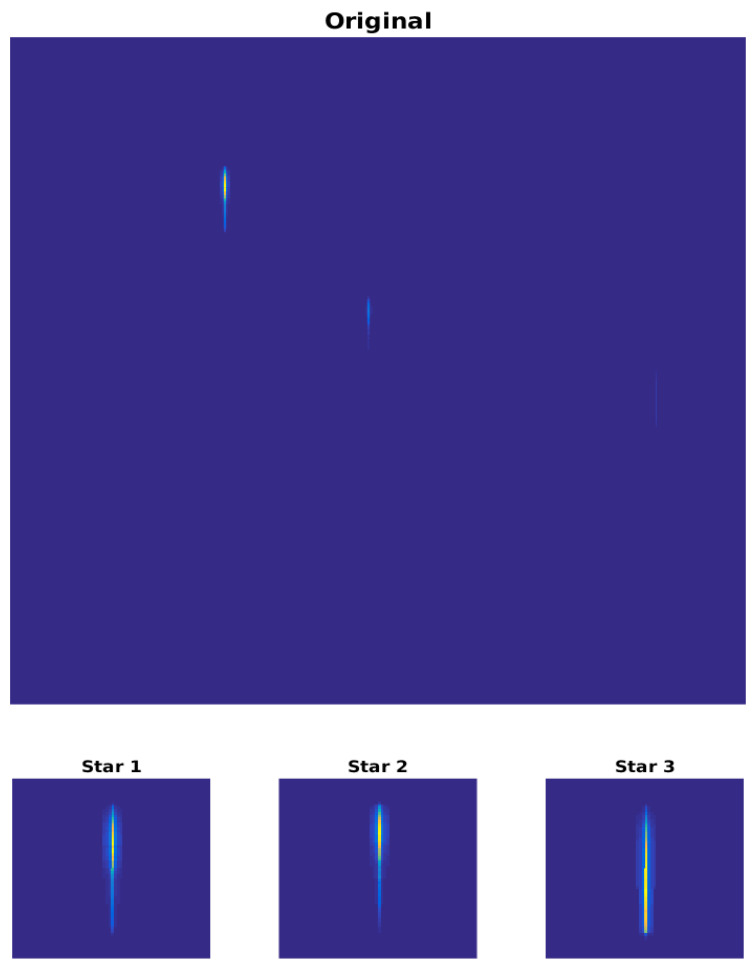
Simulated image in counts with a detail of the spectrum obtained for each of the three simulated stars.

Figure 9 shows a simulated image belonging to set#C together with a detail of the region containing the stars’ spectra.

### 5.4. Data Reconstruction

The simulated images were used as an input to the CS reconstruction algorithms to evaluate the overall system performance. The images are ordered row-wise in a one-dimensional vector x; the sensing matrix Φ has entries drawn at random from a Bernoulli distribution with probability 0.5. Indeed, it is known that random matrices are incoherent with any other fixed basis; the results reported below are obtained averaging over 100 realizations of the sensing matrix Φ and the noise. In this work, we decided to not employ a deterministic matrix Φ since we did not want to make any specific assumptions on the image data in the sensing process. The matrix Ψ is taken as the identity as the data are sparse in their original domain. Orthogonal matching pursuit (OMP) [35] was the algorithm used for the image reconstruction. While OMP does not provide reconstructions as accurate as other algorithms available in the literature, it has been selected as its complexity is low, allowing the sensing and reconstruction of the image without dividing it into tiles; moreover, OMP can be easily modified if needed, e.g., adding partial information about the signal support. Other algorithm may also be employed for reconstructing these images. With the baseline version of OMP, reconstruction of one image takes around 40 s on a computer with an Intel 4-core i7 CPU running at 2.7 GHz.

**Figure 10:**
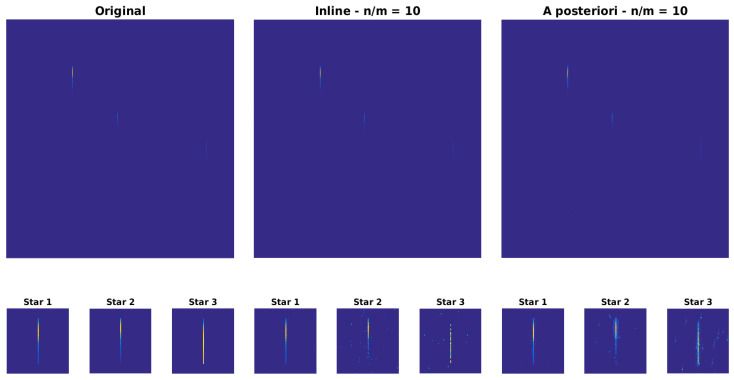
Image reconstruction (n/m = 10): comparison between the original image, the reconstructed image with inline MTF compensation, and the reconstructed image with a posteriori compensation of the MTF after the image reconstruction.

**Figure 11:**
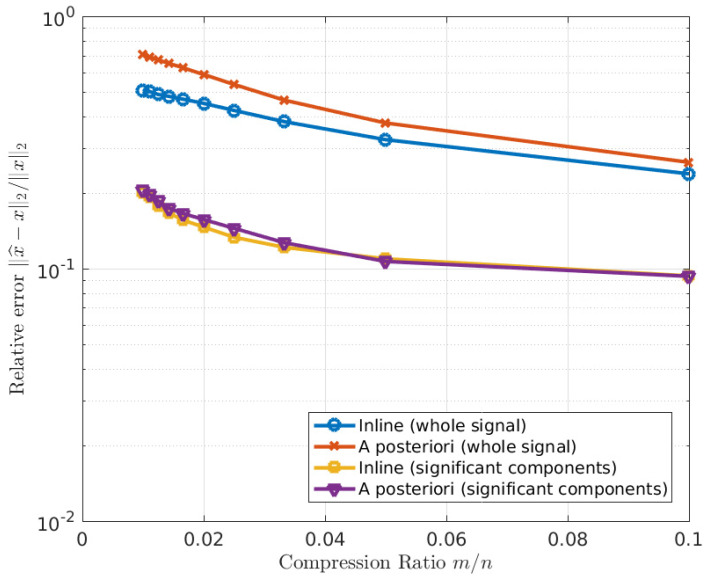
Image reconstruction: relative error as a function of relative number of measurements (CR).

**Figure 12:**
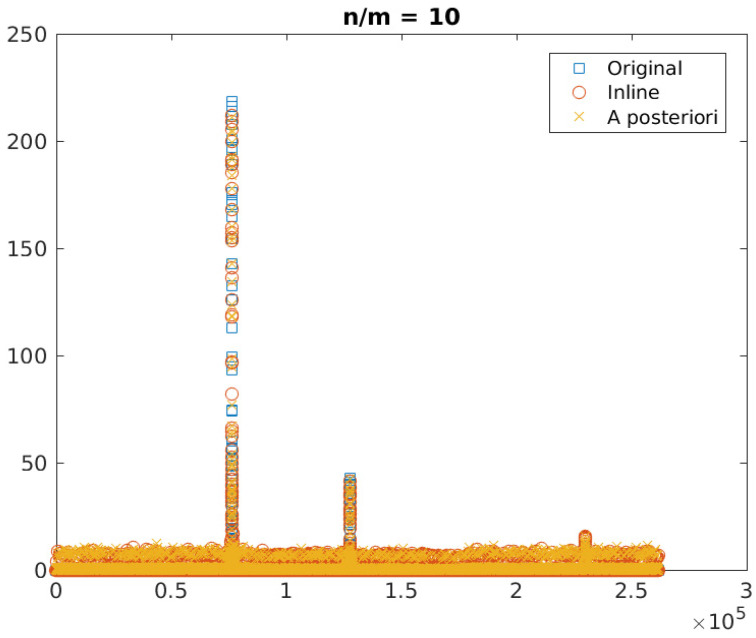
Image reconstruction (n/m = 10): comparison—in raster scan order—between the original image, the reconstructed image with inline MTF compensation, and the reconstructed image with a compensation of the MTF after the image reconstruction. The three peaks in the image represent the three stars.

Figure 10, Figure 11 and Figure 12 show the comparison between the original image, the reconstructed image with inline MTF compensation, and the reconstructed image with a posteriori compensation of the MTF after the image reconstruction, respectively. Inline MTF compensation refers to the MTF being approximately inverted directly during the CS reconstruction process, where the sensing matrix Φ is the product of an actual sensing matrix and of a degradation kernel describing the point spread function; a posteriori compensation refers to the MTF inversion being applied to the reconstructed data. We showed that having side information available about the position of the star spectra in the image significantly improves reconstruction quality, as it allows compensating the MTF affecting the significant image components, i.e., the star spectra, as can be noticed in Figure 10, Figure 11 and Figure 12.

Figure 10 and Figure 12 show the original and reconstructed data, respectively in image format and in raster scan format. Figure 11 provides a quantitative analysis of the relative reconstruction error as a function of the amount of compression. The error is reported for the whole signal (i.e., all pixels) and separately for the significant components (pixels belonging to the stars). Indeed, the error on the whole signal is larger because the algorithm attempts to reconstruct the noise in the image, which is not sparse.

Figure 12 provides a more quantitative description of the reconstructed images shown in Figure 10. The horizontal axis is the index of the image pixels read in raster scan order, whereas the vertical axis is the image pixel intensity. Pixel values are represented as circles having different color. The three peaks in the image represent the three stars, and it can be seen that the reconstructed images are radiometrically close to the correct values.

From the point of view of data reconstruction quality, the results showed good performance of the designed instrument. Quality assessment of the reconstructed data was performed by measuring several quality metrics chosen from among those recommended by the Consultative Committee for Space Data Systems (CCSDS) and by comparison with the outcomes of a CCSDS-Image Data Compression (IDC) data compressor. Table 4 reports several reconstruction metrics commonly used by the CCSDS to assess the quality of images reconstructed from lossy compression—a problem similar in spirit to CS. The metrics are the mean square error (MSE), the root MSE, the signal-to-noise ratio SNRV in dB (considering the signal power to be the variance and not the quadratic value of the signal), and the mean absolute error (MAE). As expected, all metrics improve as the ratio *m/n* increases.

**Figure 13:**
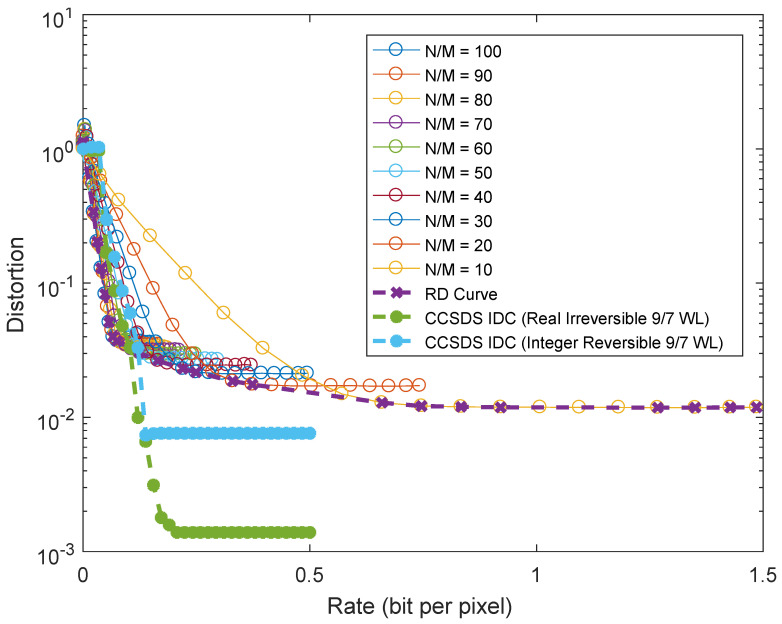
Rate-distortion analysis: distortion (MSE) of the reconstructed image as a function of rate in bits per measurement.

Finally, we performed a rate–distortion analysis of the CS system and compared it to the powerful compression algorithm in the CCSDS-IDC recommendation. The rate–distortion curve for the CS system is represented by the magenta curve in Figure 13, which corresponds to the envelope of all achievable operating points. While in principle, adaptive data compression as in the IDC recommendation is expected to outperform a universal compression method such as CS, it can be seen that there are regions corresponding to strong compression in which CS outperforms the IDC standard.

All the tested metrics showed a consistent behavior in which a lower CR corresponds to higher quality. In terms of rate-distortion tradeoff, the results obtained showed that the R-D performance of the CS instrument is satisfactory, as at high CRs, the CS instrument outperforms a conventional instrument equipped with a standard data compression board, whereas the performance loss of the CS instrument is limited at medium CRs. However, the images are affected by a relevant amount of noise, which limits the achievable quality of the CS instrument. Anyway, it should be noted that this significant amount of noise would affect also the performance of a conventional instrument. In the high CR regime, these results are expected to translate into a downlink gain with respect to a standard tool, even if equipped with a compression board. In reality, the designed instrument can achieve CRs of 20 and even higher without a significant loss of information (Figure 11). Our aim is to recover the image compensating the effects of MTF and noise, neglecting other effects, such as the quantum efficiency or the transmittance. Without any knowledge of the star positions, we can integrate in the reconstruction algorithm the compensation of the MTF affecting the image background (inline compensation). 

## 6. Mechanical Assembly and Budgets Assessment

In order to provide an overall assessment of the payload, we studied a possible arrangement of the proposed instrument in a suitable mechanical assembly. We also performed a preliminary evaluation of the expected budgets in terms of the mass, size, and power consumption of the instrument.

On the basis of the optical CAD implemented for the proposed instrument, and the experience gained in the assembly of similar payloads by Leonardo Company as a partner of the project, an overall preliminary budget assessment for the mechanical arrangement was made as follows:

- Optical head (OH): 870 mm × 642mm × 507 mm; 25.4 kg (including contingency 20%);
- Front-end electronics (FEE): 200 mm × 140 mm × 80 mm; main electronics (ME): 240 mm × 200 mm x 80 mm, total mass: 5 kg (including contingency 20%);
- Power estimate: less than 30 W (assumptions: DC/DC Efficiency = 9.75; contingency 20%).

**Figure 14:**
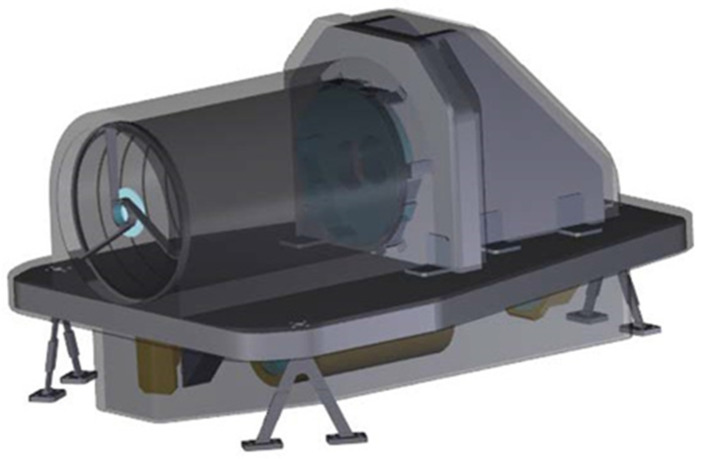
Overview of the mechanical assembly of the instrument.

The mechanical arrangement of an elegant breadboard of the proposed instrument is shown in Figure 14. The mechanical arrangement is organized in two levels: the collection optics are placed in the upper part of the optical breadboard, while the spectrometer with the DMD, the detection unit, and the electronics are placed in the lower part.

## 7. Discussion

The proposed instrument implements a CS architecture based on the single-pixel camera principle applied to provide slitless spectroscopy data for space applications. Presently, there are not studies addressing this implementation of the CS approach and single-pixel camera principle. The instrument’s architecture uses a COTS DMD as a core element to apply the CS modulation mask to the image of the observed scene. Whenever a high-resolution sensor is unavailable (or too expensive), a CS instrument based on the single-pixel camera principle has an intrinsic advantage over traditional instruments using array detectors. Although with a limited flexibility with respect to ISSIS or STIS, the proposed instrument will have reduced budgets in terms of mass and cost—yet increased complexity in the instrument’s architecture—while the intrinsic sparseness of the data would allow for less demanding requirements in terms of downlink bandwidth. In this study, we showed that the CS approach allowed a drastic reduction of the number of coded acquisitions. The high sparsity of the acquisition domain showed good potential for obtaining good-quality data reconstruction with a high compression ratio (CR).

From an optical point of view, a critical issue can arise from diffraction effects due to the dimensions of micro-mirrors: while the UV-Vis spectral range—as in this study—does not pose any issue, SLM diffraction effects cannot be neglected when dealing with longer wavelengths. In such cases, the Airy disk diameter associated with the image on SLM plane may need to be reduced by reducing the F number of the imaging optics, thus increasing the difficulties (and the cost) of optics manufacturability. A large F number implies a higher number of optical aberrations to be handled, leading to the need of a larger number of optical surfaces and/or aspherical (and in general freeform) optical elements.

From the point of view of data reconstruction quality, the results showed a good performance of the designed instrument. Quality assessment of reconstructed data was performed by measuring several quality metrics chosen from among those recommended by CCSDS and by comparison with the outcomes of CCSDS-IDC data compressor. All the tested metrics showed consistent behavior, in which a lower CR corresponds to higher quality. In terms of rate-distortion (R-D) tradeoff, the results obtained showed that at high CRs, the R-D performance of the CS-based instrument is better than that of a conventional instrument equipped with a standard data compression board. However, the images are affected by a relevant amount of noise, which limits the achievable quality of the CS-based instrument. Regardless, it should be noted that this significant amount of noise would also affect the performance of a conventional instrument, which, however, could not achieve the same compression level. These results also envisage a downlink gain with respect to a standard tool even if equipped with a compression board. In reality, the designed instrument can achieve CRs of 20 and even higher without a significant loss of information.

From the point of view of the reconstruction algorithms, we successfully tested the possibility of compensating the instrumental response of the sensor together with the reconstruction of the data. Since both CS reconstruction and MTF compensation are inverse problems, they can be carried out jointly. This represents a considerable advantage since it permits us to skip a step that is typically required for processing data from spectroscopic observations of the sky. The MTF compensation also yielded a slight improvement (a few percent, at least) in the quality of the reconstructed data (at the same CR). 

Concerning the instrument budgets, the main advantage of the CS-based instrument is that it does not need a data compression board. This offers an advantage in terms of power consumption, so the power absorbed by the CS instrument is less (about 15 W, typically) than that of the corresponding traditional one, and in terms of mass, although to a limited extent (about a 1 kg decrease with respect to the overall instrument’s mass). The lack of a compression board also prevents the need to implement an ad hoc compression algorithm along with the development costs of an electronic board. Since presently, there are not hardware implementations of data compression algorithms other than Joint Photographic Experts Group (JPEG), this represents a significant advantage. On the other hand, the volume of the instrument does not show a significant decrease: despite the possibility of replacing an array detector with a single element detector, the optical components that have the major impact on the instrument’s volume, such as the entrance optics and the spectrometer, have the same size as in the corresponding traditional instrument. This aspect is essential to achieve a good radiometric signal quality. In addition, a CS-based instrument requires a double system of optics: the imaging optics (used to focus the image at the SLM field stop) and the condenser optics (used to integrate the spatially coded image). The need of the SLM as core element adds a further increase in the complexity of the instrument, while the specific use of a DMD adds some constraints to the optical design because of being a reflective mask and having a limited angle of reflection. These issues increase the requirements on optics.

Concerning the technology readiness level of SLM as a key enabling technology for CS instruments, presently there are not space-qualified DMD models, although intense space qualification activity is ongoing both at the National Aeronautics and Space Administration (NASA), in collaboration with the Rochester Institute of Technology and Johns Hopkins University [36], and European facilities on one Texas Instruments model for future missions [37]. A drawback of DMD models is that these devices work only in reflection mode and that only a limited range of micromirrors dimensions is available, the size typically being overdimensioned for the UV-Vis range and under-dimensioned for MWIR-LWIR operation. The use of other SLM types, such as liquid crystal on silicon (LCOS) or microshutter array (MSA), is presently still limited by the low modulation speed, small number of elements, and lower suitability for space qualification. However, since the interest in the development of this type of devices is increasing swiftly—as is also demonstrated by various ongoing studies conducted by international research laboratories and large companies—a substantial technological development is expected in this field in the next years.

## 8. Conclusions

In this paper, we presented the instrumental concept and optical design of a CS-based imaging spectrometer for UV–Vis stellar spectroscopy, and we discussed its expected performances in terms of data reconstruction quality. The instrument’s concept relies on the use of a COTS DMD for implementing a CS single-pixel camera architecture, which is used to provide slitless spectroscopic images of stars. The optical design led to a pixel-limited instrument in the entire 300 nm–650 nm spectral range. The spectral sampling ranges from 2.2 nm @ 300 nm to 22 nm @ 650 nm with a total of 50 samples for each spectrum.

Data reconstruction showed good performance measured by several quality metrics chosen among those recommended by CCSDS. The designed instrument can achieve CRs of 20 and even higher without a significant loss of information. The inherent compression of data, intrinsic to CS approach, also implies that the compression board and the development of an ad hoc compression algorithm is no longer needed. On the whole, the study permitted us to gain in-depth knowledge of the CS technology and its applicability to instrumentation specifically designed for space applications.

## Figures and Tables

**Table 1:** STIS spectroscopic data: sparsity level in the original and transform domains (scale is between 0 and 1).

	Sparsity Level Pixel Values	Sparsity Level 2D-DCT Coefficients
Without Threshold	0.79	0.72
With Threshold	2.29 × 10^-4^	0.72

DCT = discrete cosine transform.

**Table 2:** Optical design of the instrument: optical components and relevant materials.

Parameter	Value
System focal length	2321 mm
Field of view (FOV)	0.273°
Primary mirror	Parabolic mirror
Primary mirror diameter	235 mm
F#	9.88
Secondary mirror	Hyperbolic mirror (conic const. = −2.7)
Spectrometer lens system	2 symmetric spherical lens systems
Effective focal length of a single system of lenses	723.5 mm
Back focal length	494.4 mm
Spectrometer prism angle	2.70°
Spectrometer resolving power	136@300 nm; 29.5@650 nm
Prism material	FUSED SILICA
Arm inclination (after prism)	1.318°
SLM type	DMD
Model, provider	DLP^®^9500UV, Texas Instruments Inc.
Material	Aluminum, UV-coated
Spectral range	300 nm–650 nm
Reflectivity	88%
Array diffraction efficiency	86%
Array fill factor	92%
Surface	Micromirrors
Orientation	Orthogonal
Pixel pitch	10.8 micron
Number of micromirrors (array size)	1920 × 1080
Area of interest	1024 × 1024 micromirrors (we use 2 × 2 binned micromirrors)
Tilt angle	+/−12°
Micromirror rotation axis	Diagonal
DMD maximum frame rate	23,148 Hz
DMD size (length × height × width)	42.164 mm × 42.164 mm × 7.03 mm
Used area dimensions (length × height)	11.30 mm × 11.30 mm
Condenser lens material	FUSED SILICA
Condenser lens diameter	32 mm
Condenser transmission	99%
Focal length	38 mm
Distance from DMD	60 mm
Distance from the detector sensing area	36.4 mm
Surface	Spherical
Detector type	Photomultiplier module (PMT)
Model, provider	Hamamatsu, H10682 model
Material	Ultra Bialkali cathode
Pulse pair resolution	20 ns
Spot diameter on single-pixel sensor	4.2 mm
Sensing area (diameter)	8 mm

**Table 3:** List of spectrometers optical elements materials.

Optical Element	Value
Prism angle	2.7°
Prism material	Fused silica
Lens 1, 7	CaF_2_
Lens 2, 8	MgF_2_
Lens 3, 9	Fused silica
Lens 4, 10	Fused silica
Lens 5, 11	MgF_2_
Lens 6, 12	Quartz

**Table 4:** Reconstruction results using CCSDS metrics.

C.R. [%]	Algorithm	MSE	RMSE	SNRV	MAE
0.010	Inline	0.73	0.85	5.92	49.08
	A Posteriori	1.41	1.19	3.06	61.55
0.011	Inline	0.72	0.85	5.97	46.00
	A Posteriori	1.36	1.16	3.22	57.85
0.013	Inline	0.68	0.83	6.20	42.13
	A Posteriori	1.28	1.13	3.46	53.06
0.014	Inline	0.66	0.81	6.34	39.40
	A Posteriori	1.21	1.10	3.73	49.02
0.017	Inline	0.63	0.79	6.58	36.49
	A Posteriori	1.11	1.05	4.09	43.77
0.020	Inline	0.58	0.76	6.90	33.22
	A Posteriori	0.98	0.99	4.61	39.67
0.025	Inline	0.51	0.72	7.46	30.79
	A Posteriori	0.82	0.91	5.40	34.16
0.033	Inline	0.42	0.64	8.35	28.82
	A Posteriori	0.62	0.78	6.65	30.19
0.050	Inline	0.30	0.55	9.78	27.44
	A Posteriori	0.41	0.64	8.45	27.57
0.100	Inline	0.16	0.40	12.48	25.28
	A Posteriori	0.20	0.45	11.57	24.90

## Data Availability

Data available on request to the authors.
